# Clinical simulation and deliberate practice in rapid-cycle deliberate practice: a randomized clinical trial

**DOI:** 10.1590/0034-7167-2024-0632

**Published:** 2025-12-12

**Authors:** Gisele Cabral da Silva, Cássia Regina Vancini Campanharo, Maria Carolina Barbosa Teixeira Lopes, Meiry Fernanda Pinto Okuno, Carla Roberta Monteiro Miura, Ruth Ester Assayag Batista

**Affiliations:** I Universidade Federal de São Paulo. São Paulo, São Paulo, Brazil

**Keywords:** Simulation Training, Stroke, Clinical Competence, Emergency Nursing, Patient Simulation., Entrenamiento Simulado, Accidente Cerebrovascular, Competencia Clínica, Enfermería de Urgencia, Simulación de Paciente.

## Abstract

**Objectives:**

to compare clinical simulation with a standardized patient with rapid-cycle deliberate practice in knowledge acquisition, perception of experience and scenario design in the care of patients with suspected stroke.

**Methods:**

a randomized clinical trial conducted between 2022 and 2023 involving 134 undergraduate nursing students. After a theoretical class, participants were randomly assigned to one group. A questionnaire was administered before and after the practical sessions to assess knowledge, in addition to the Educational Practices Questionnaire and the Simulation Design Scale.

**Results:**

the groups showed significant improvement in knowledge (p<0.001), with no difference between them (p=1.0). Rapid-cycle deliberate practice obtained a better assessment regarding design (p=0.03).

**Conclusions:**

the strategies are effective and recommended in nursing education, although rapid-cycle deliberate practice showed advantages in student engagement. Universal Trial Number: U1111-1295-1246.

## RESUMEN

**Objetivos:**

comparar la simulación clínica con paciente estandarizado con la práctica deliberada en ciclos rápidos en la adquisición de conocimientos, en la percepción de la experiencia y diseño del escenario, y en la atención de pacientes con sospecha de accidente cerebrovascular.

**Métodos:**

ensayo clínico aleatorizado realizado entre 2022 y 2023 con 134 estudiantes de enfermería de pregrado. Tras una clase teórica, los participantes fueron asignados aleatoriamente a un grupo. Se administró un cuestionario antes y después de las sesiones prácticas para evaluar los conocimientos, además del Cuestionario de Prácticas Educativas y la Escala de Diseño de Simulación.

**Resultados:**

los grupos mostraron una mejora significativa en el conocimiento (p<0,001), sin diferencias entre ellos (p=1,0). La práctica deliberada en ciclos rápidos obtuvo una mejor evaluación en cuanto al diseño (p=0,03).

**Conclusiones:**

las estrategias son eficaces y recomendables en la formación en enfermería, aunque la práctica deliberada en ciclos rápidos mostró ventajas en la participación estudiantil. *Universal Trial Number:* U1111-1295-1246.

## INTRODUCTION

The approach to teaching in healthcare has undergone global transformations. Educational institutions seek to create and apply learning methods based on the constant interaction between theory and practice, encouraging creativity, and promoting meaningful learning^(1)^. Both competence and performance are the result of a combination of various technical and human skills that students/professionals need to improve their daily work with patients. Competence is related to what students or professionals accomplish in controlled situations, while performance is associated with their actions during actual practice^(2)^.

Clinical simulation is an active educational approach that uses realistic scenarios to promote learning. This teaching methodology is effective in reinforcing knowledge, ensuring patient safety, and reducing the risk of iatrogenic errors. At the end of the scenario, a debriefing takes place, contributing to learning consolidation^(1)^. In 2014, different approaches emerged, such as rapid-cycle deliberate practice (RCDP). This innovative methodology consists of dividing the simulated clinical case into cycles, which students repeat until the necessary competence is acquired^(3-5)^. Feedback is provided in cycles, using a teaching method with quick pauses to correct errors and reinforce learning based on student performance^(6,7)^. It is important to emphasize the variation in the method for reinforcing acquired knowledge. In clinical simulation, debriefing is used to analyze situations during the scenario’s execution and encourage improvements without interrupting the activity. In RCDP, feedback is provided at the end of each scenario cycle, allowing students to practice and improve their skills until they reach the desired level within each cycle^(8-11)^.

Clinical simulation is an excellent learning tool because it provides early practical experience of nursing, allowing students to combine theory learned in the classroom with clinical practice. This contributes significantly to knowledge retention, in addition to improving skill development, leadership, decision-making, and teamwork^(10-12)^. Studies have also shown that RCDP contributes to increased knowledge and self-confidence, and improved skills of individuals^(13-16)^.

During their training in healthcare, many students struggle to gain practical experience in various clinical situations, including treating patients with suspected stroke. In Brazil, in 2020, 99,010 deaths from this disease were recorded, and in 2022, it returned to being the leading cause of death in the country^(17)^.

Clinical simulation and RCDP are techniques that can help improve the skills needed to manage patients presenting with signs and symptoms of stroke. Stroke can lead to death or leave physical and mental health consequences, restricting daily activities, impairing quality of life and efficiency, and resulting in additional healthcare costs due to complications^(18)^. Furthermore, it is one of the leading causes of death and disability, and given its economic and social impact, it is essential to train students in the early identification of signs and symptoms to ensure a favorable outcome for patients. Treatment is time-dependent, meaning early identification is essential for a more favorable outcome.

In this context, a high-fidelity scenario is recommended for training students in stroke recognition and implementation of initial actions. Developing this competence is complex, as it involves decision-making, problem-solving, communication, and teamwork^(17,18)^, making it challenging for educators to choose the best pedagogical strategy to achieve the proposed activity objectives. Therefore, this study is considered important because it supports facilitators’ choices, enabling more assertive and effective planning.

## OBJECTIVES

To compare clinical simulation with RCDP in knowledge acquisition, perception of experience and scenario design in the care of patients with suspected stroke.

## METHODS

### Ethical aspects

This research was approved by the *Universidade Federal de São Paulo* Research Ethics Committee. In accordance with Brazilian Resolution 466/2012, all ethical and legal standards regarding research involving human subjects were respected, and written informed consent was obtained from all individuals involved in the study. The clinical trial was approved under number U1111-1295-1246 in the Brazilian Clinical Trials Registry.

### Study design, period and location

This is a randomized, prospective, controlled clinical trial, with random allocation, following the descriptions according to the CONsolidated Standards Of Reporting Trials^(19)^. Data collection took place between 2022 and 2024, and was carried out at the simulation center of a public university, located in the city of São Paulo, SP, Brazil.

### Population, inclusion and exclusion criteria

All undergraduate students regularly enrolled in the final year of their undergraduate nursing program in 2022 and 2023 were invited. Students who participated in the 40-minute lecture on nursing care for patients with suspected stroke in the emergency department were included. Students absent on the day of data collection were excluded.

### Study protocol

The researcher applied the simulation with standardized patient (SWSP) and RCDP to participants based on a validated scenario for treating patients with suspected stroke in the emergency department. The scenario involved treating an adult patient in the emergency room with symptoms suggestive of stroke. The 65-year-old male patient arrived at the emergency department with difficulty walking, hemiparesis, and slowed verbal communication. The groups were expected to begin assessing patients’ symptoms and history, provide appropriate guidance, administer scales, monitor, and collect blood tests, concluding with referral for imaging. The SWSP group had ten minutes to develop the scenario, and at the end, positive points and areas for improvement were identified according to guidelines (debriefing - suggested 20 to 30 minutes), while the RCDP group took an average of 60 to 70 minutes to complete the cycles. The RCDP scenario was divided into four cycles, which addressed the expected objectives for each phase. Corrective actions and suggestions for better behavior were made during the cycles and influenced the decision to allow students to complete all cycles (feedback)^(20)^.

To ensure student allocation, numbered, opaque, and sealed envelopes were prepared. Half of the envelopes contained a piece of paper describing the SWSP group, and the other half contained the RCDP group description. These envelopes were placed inside folded cardboard to ensure the text was invisible to all participants and the researcher, who conducted the simulation in all groups. All envelopes were placed in a frosted box with an opening in the top lid, which participants could remove before beginning the simulated activity^(21)^. In the SWSP group, they were further subdivided into two groups: simulation acting (students perform the service in the scenario); and simulation observing (students observe the group that performs the service in the scenario).

The knowledge assessment instrument was validated by a group of experts and administered before and after the two educational strategies. It consisted of nine objective questions, with four possible answers, and only one correct answer. The questions addressed signs and symptoms, risk factors, assessment scales, and specific care for patients with suspected stroke.

Immediately after the simulated practices (SWSP and RCDP), in order to assess students’ opinion about the scenario and experience, the Simulation Design Scale and the Educational Practices Questionnaire were applied (both validated for Brazilian Portuguese)^(22,23)^. The response pattern for both questionnaires was a five-point Likert type, corresponding to: 1 - Strongly disagree with the statement; 2 - Disagree with the statement; 3 - Undecided; 4 - Agree with the statement; 5 - Strongly agree with the statement.

### Analysis of results and statistics

A descriptive statistical analysis (mean, minimum, maximum, and standard deviation) was performed on the Simulation Design Scale and Educational Practices Questionnaire results, as well as on each item and its corresponding factors. For statistical analysis, the database was structured in Python 3.9 using the Pandas library. The Shapiro-Wilk test was performed, identifying a lack of normality; therefore, nonparametric tests were used. The Wilcoxon test was used for comparability between paired groups before and after the approach method (mean number of correct answers in the preand post-test and comparison of the groups’ responses to the Educational Practices Questionnaire) after the educational intervention, using the “less” hypothesis. The Mann-Whitney U test was used to compare the different educational strategies. The significance level was set at 5%.

## RESULTS

A total of 134 regularly enrolled fourth-year nursing students were included. Students were randomly assigned to two groups: 66 students in the SWSP group and 68 students in the RCDP group. In the SWSP group, students were redistributed into two subgroups: 33 students in the practicing group and 33 in the observing group. Regarding knowledge, there was an improvement in the mean post-activity scores compared to pre-test scores, regardless of the strategy used: RCDP or SWSP (P<0.001) (Table 1).

**Table 1:** Comparison of the mean number of correct answers in the preand post-test between the simulation with standardized patient and rapid-cycle deliberate practice groups in the preand post-test, São Paulo, São Paulo, Brazil, 2023 (N=134)

	Pre-test		Post-test		*p* value^*^
	Mean (standard deviation)	Minimum- Maximum	Mean (standard deviation)	Minimum- Maximum	
Simulation with standardized patient group (n=66) rapid-cycle deliberate practice group (n=68)	7.76 (1.28) 7.96 (1.21)	3.00 - 9.00 3.00 - 9.00	8.44 (0.84) 8.66 (0.63)	5.00 - 9.00 7.00 - 9.00	p<0.001 p<0.001

* *Probability of significance using the Wilcoxon test.*

When comparing the increase in knowledge in the preand post-test, regardless of the strategy used, the RCDP group showed a gain of 51.47% in the sum of correct answers through the questionnaire applied to assess knowledge immediately after the simulated practice (post-test), while the SWSP group had an increase of 46.97%, i.e., there was a statistically significant improvement (p < 0.001) as a result of the post-test of the two simulation strategies. However, when comparing the two strategies, there was no difference between the groups (p=1.0) (Table 1).

There was an improvement in participants’ knowledge between the assessed moments (pre-test and post-test), regardless of the allocation in the group, acting in simulated care with the use of a standardized patient (n=33) or in the observing group (n=33) (p<0.001). The best mean knowledge was that of the simulation acting group (8.64) (Table 2). However, when comparing the two groups (acting and observing), there was no statistical difference between them (p=0.76).

**Table 2:** Comparison of the mean number of correct answers in the preand post-test between the groups (simulation acting and simulation observing), São Paulo, São Paulo, Brazil, 2023 (N=66)

	Pre-test		Post-test		*p* value^*^
	Mean (standard deviation)	Minimum- Maximum	Mean (standard deviation)	Minimum- Maximum	
Simulation in action (n=33) Simulation observing (n=33)	7.97 (1.17) 7.55 (1.35)	4.00 - 9.00 3.00 - 9.00	8.64 (0.59) 8.24 (0.99)	7.00 - 9.00 5.00 - 9.00	p<0.001 p<0.001

* *Probability of significance using the Wilcoxon test.*

In relation to the analysis of the Simulation Design Scale and Educational Practices Questionnaire, the two educational strategies presented a mode of 5 and means above 4.2 for all factors, as well as for the scales as a whole, demonstrating a very positive assessment by students for both strategies (Tables 3 and 4).

**Table 3:** Comparison of the responses of simulation with standardized patient and rapid-cycle deliberate practice groups to the Educational Practices Questionnaire, São Paulo, São Paulo, Brazil, 2023 (N=134)

Factors	SWSP group					RCDP group					*p* value^¶^
	Mean	SD	Min.	Max.	Mode	Mean	SD	Min.	Max.	Mode	
Active learning	4.74	0.51	2	5	5	4.80	0.44	1	5	5	p=0.02
Collaboration	4.64	0.73	2	5	5	4.36	0.92	1	5	5	p=0.004
Diverse ways of learning	4.68	0.49	3	5	5	4.80	0.39	4	5	5	p=0.03
High expectations	4.72	0.56	2	5	5	4.86	0.34	4	5	5	p=0.05
Overall mean	4.72	0.54	2	5	5.00	4.75	0.53	1	5	5.00	p=0.05

*SWSP - simulation with standardized patient; RCDP - rapid-cycle deliberate practice; SD - standard deviation; Min - minimum; Max - maximum; ¶p value - probability of significance using the Mann-Whitney U test.*

**Table 4:** Comparison of responses from simulation with standardized patient and rapid-cycle deliberate practice groups regarding the Simulation Design Scale, São Paulo, São Paulo, Brazil, 2023 (N=134)

Factors	SWSP group					RCDP group					*p* value^¶^
	Mean	SD	Min.	Max.	Mode	Mean	SD	Min.	Max.	Mode	
Objectives and information	4.79	0.44	2	5	5	4.83	0.40	2	5	5	p=0.13
Support	4.68	0.63	2	5	5	4.84	0.41	2	5	5	p<0.001
Problem-solving	4.73	0.52	2	5	5	4.82	0.43	1	5	5	p=0.02
Feedback/guided reflection	4.87	0.37	2	5	5	4.89	0.30	4	5	5	p=0.67
Fidelity (realism)	4.54	0.61	2	5	5	4.45	0.69	2	5	5	p=0.33
Overall mean	4.75	0.51	2	5	5.00	4.80	0.45	1	5	5.00	p=0.03

*SWSP - simulation with standardized patient; RCDP - rapid-cycle deliberate practice; SD - standard deviation; Min - minimum; Max - maximum; ¶p value - probability of significance using the Mann-Whitney U test.*

In the Educational Practices Questionnaire, the RCDP group received higher scores on the active learning factor (p=0.02), meaning the items opportunity to reflect on receiving clues during the simulated practice, discussing objectives and ideas with the facilitator, reflecting on and learning from the facilitator’s comments, and learning time were more productive. On the collaboration factor, the SWSP strategy received higher scores, meaning participants had more opportunities to work with peers during the simulated practice and complete the work together (p=0.004). As for the diverse ways of learning factor, the RCDP group rated it more positively, as they felt the practice offered a different approach to contribute to their learning. On its final factor, which addresses high expectations, the RCDP received higher scores, as participants reflected that the objectives were clear and easy to understand, and that the facilitator communicated the objectives to be achieved (p=0.05) (Table 3).

In relation to the Simulation Design Scale, the RCDP strategy was best assessed in relation to support, which refers to when the participant had their needs recognized during the simulated activity (p<0.001), and problem-solving, which assesses the opportunities to solve the problem situations identified in the scenario (p=0.02). Furthermore, in the scale application result as a whole, the RCDP strategy was best assessed by students (p=0.03) (Table 4).

## DISCUSSION

### Simulation with standardized patient and rapid-cycle deliberate practice

Undergraduate nursing education has sought innovative educational strategies for acquiring professional skills^(24,25)^. Studies confirm this finding, demonstrating that simulation is beneficial for building knowledge in a controlled and safe way, as well as for developing problem-solving skills, and provides opportunities for receiving feedback on one’s performance, which contributes to improving one’s performance^(22,23)^. There is also evidence that RCDP increases levels of knowledge and self-confidence, improving skills, which enhances practice^(26,27)^.

A study^(28)^ conducted with 58 nursing undergraduate students, which assessed the effectiveness of clinical simulation in teaching bed bathing, demonstrated that students who participated in the simulation performed better in the immediate and delayed post-test. Another experimental study on medication administration, conducted with 85 nursing students, using a preand post-test method, obtained similar results^(29)^.

Studies have demonstrated the effectiveness of RCDP for training in cardiopulmonary resuscitation, resulting in higher quality compressions, faster time to initiate compressions and defibrillation, even compared to training conducted by the American Heart Association^(30-32)^.

The results were similar to another study conducted in Rwanda, which compared the two strategies, in which 51 pediatric residents were randomly assigned to the RCDP or traditional simulation group. There was a 21% increase in preand post-test performance in both groups, but there was no difference between groups^(33)^.

However, a randomized study of 76 physicians from the Graduate Program in Emergency Medicine at *Hospital Israelita Albert Einstein* found conflicting results when comparing participants’ performance in clinical simulation and RCDP during cardiopulmonary resuscitation. The RCDP strategy was associated with a significantly higher chest compression fraction and significantly shorter time between rhythm recognition and defibrillation^(34)^.

### Simulation acting and simulation observing

The results of a systematic review that included nine articles found that the observer’s role in simulation had learning outcomes equal to or even better than those who acted in the scenarios^(35,36)^. This phenomenon can be explained by the principles of vicarious learning, described by Bandura^(37)^, according to which individuals can acquire new knowledge, skills, and attitudes by observing the behavior of others, without the need to directly experience the experience. Another study that assessed student satisfaction concluded that there was no significant difference between the roles played in the simulation (active or observer)^(38)^.

Conducting simulation scenarios with methodological rigor, valuing each stage of the process and including all participants, regardless of their role, from briefing to debriefing, is essential to ensuring the most effective learning possible. This finding confirms the results of a systematic review of nine articles, which found that observers in simulations produced learning outcomes that were equal to or even better than those who participated in the scenarios^(35,36)^. Another study that assessed student satisfaction concluded that there was no significant difference between the roles played in simulation (active or observer)^(38)^.

Conducting simulation scenarios with methodological rigor, valuing each stage of the process and including all participants, regardless of their role, from the briefing to the debriefing, is essential to ensure that learning occurs as effectively as possible.

### Educational Practices Questionnaire and Simulation Design Scale

In the Educational Practices Questionnaire, RCDP was better assessed in three factors, but was only worse in the collaboration factor, which can be justified by the fact that, in the RCDP strategy, students performed care for patients with suspected stroke individually and, in the SWSP group, they performed care in pairs.

Educational activities are present in teaching approaches and techniques^(39)^. Effective educational practices involve following guidelines that contribute to learning, such as encouraging student-teacher interaction, promoting student cooperation and active learning, providing immediate feedback, dedicating time for study, setting high expectations, and respecting students’ abilities and ways of learning^(40)^.

For learning to be effective, it is essential that students have the chance to discuss and expand their knowledge based on their previous learning - concepts, experiences, among others - and share them with the facilitator, other students and collaborators^(41)^. Students’ active participation in simulated scenarios allows them to critically analyze and assess various practical situations. This reflection is essential for improving their professional practice^(41,42)^. Furthermore, the more actively a student participates in the learning process, the better they are able to assimilate the information provided^(42)^; this is evidenced by students’ assessment of active learning factors and high expectations regarding the RCDP strategy. Student participation in the process of sharing the listed objectives is important, highlighting the importance of the teaching and learning interface. Concerning participants’ behavior (facilitator and students), they are expected to demonstrate attributes of confidentiality, compassion, commitment, collaboration, honesty, mutual respect, and involvement in the teaching and learning process^(41)^.

The analysis of the design of simulated practices highlights the need for improvements that enhance the effectiveness of the scenario as a teaching-learning strategy. In this process, the facilitator’s role is not only relevant but essential for knowledge construction. The literature^(39-42)^ indicates that the facilitator’s role transcends the operational function of conducting the simulation, assuming a strategic role in the development of critical thinking, clinical reasoning, professional judgment, and translation of theoretical knowledge into practical care. From a constructivist perspective, the facilitator acts as a catalyst for meaningful learning by promoting reflection, questioning, and providing targeted feedback, which encourages student leading role in the learning process.

### Study limitations

Limitations of this study include conducting the tests in a single simulation center and nursing students’ exclusive participation. Furthermore, although there was an objective checklist to guide the assessment of expected actions in the scenarios (SWSP and RCDP), the researcher’s own application of these strategies may have introduced bias.

### Contributions to nursing, health, or public policy

Positive assessment of both strategies demonstrates that the use of active methodologies in healthcare, even in institutions with limited resources, can facilitate learning and increase the chances of early recognition and treatment of patients with suspected stroke. This contributes to better clinical outcomes and reduced costs for the healthcare system. The study reinforces that both SWSP and RCDP are effective strategies for developing nursing competences. RCDP also offers specific advantages in student engagement and pedagogical design, important aspects for curricular decisions.

## CONCLUSIONS

RCDP and SWSP proved to be equally effective strategies for improving knowledge about initial care for stroke victims. Based on students’ assessment of the simulated experience using the Simulation Design Scale, the RCDP strategy received a better rating compared to the SWSP group (p=0.03).

When comparing the SWSP subgroups (acting and observing), there was no difference in knowledge acquisition. This indicates that, regardless of the position (acting or observing), both roles were effective in improving knowledge about initial stroke victim care.

## Data Availability

The research data are available within the article.
