# Supplementary material for: Clinical simulation and deliberate practice in rapid-cycle deliberate practice: a randomized clinical trial
Source: Rev Bras Enferm. 2025 Dec 12;78(Suppl 4):e20240632. doi: 10.1590/0034-7167-2024-0632 (PMC12707768; doi:10.1590/0034-7167-2024-0632)
Supplement: Translation

# Translation: Simulação clínica e prática deliberada em ciclos rápidos para a competência clínica: ensaio clínico randômico

**DOI:** 10.1590/0034-7167-2024-0632pt

**Authors:** Gisele Cabral da Silva, Cássia Regina Vancini Campanharo, Maria Carolina Barbosa Teixeira Lopes, Meiry Fernanda Pinto Okuno, Carla Roberta Monteiro Miura, Ruth Ester Assayag Batista

**Affiliations:** I Universidade Federal de São Paulo. São Paulo, São Paulo, Brasil

**Keywords:** Treinamento por Simulação, Acidente Vascular Cerebral, Competência Clínica, Enfermagem em Emergência, Simulação de Paciente.

## Abstract

**Objetivos:**

comparar a simulação clínica com paciente padronizado com a prática deliberada em ciclos rápidos na aquisição de conhecimento, na percepção sobre a experiência e *design* do cenário, e no atendimento ao paciente com suspeita de acidente vascular cerebral.

**Métodos:**

ensaio clínico randômico, realizado entre 2022 e 2023, envolvendo 134 estudantes do curso de bacharelado em enfermagem. Após aula teórica, os participantes foram divididos aleatoriamente entre os grupos. Aplicou-se questionário antes e depois das práticas para avaliar o conhecimento, além do Questionário de Práticas Educacionais e da Escala de *Design* da Simulação.

**Resultados:**

os grupos mostraram melhora significativa no conhecimento (p<0,001), sem diferença entre eles (p=1,0). A prática deliberada em ciclos rápidos obteve melhor avaliação quanto ao *design* (p=0,03).

**Conclusões:**

as estratégias são eficazes e recomendadas na formação em enfermagem, embora a prática deliberada em ciclos rápidos tenha apresentado vantagens no engajamento do estudante. *Universal Trial Number:* U1111-1295-1246.

## INTRODUÇÃO

O modo de abordar o ensino na área da saúde passou por transformações em âmbito global. As instituições de ensino buscam criar e aplicar métodos de aprendizado baseados na interação constante entre teoria e prática, no estímulo à criatividade e na promoção de uma aprendizagem significativa^(1)^. Tanto competência quanto desempenho são resultado de uma combinação de diversas habilidades técnicas e humanas que os estudantes/profissionais precisam possuir para aprimorar sua atuação diária com os pacientes. Competência está relacionada ao que o estudante ou profissional realiza em situações controladas, enquanto que o desempenho está associado às suas ações durante a prática real^(2)^.

A simulação clínica é uma abordagem educacional ativa que utiliza cenários realistas para promover a aprendizagem. Essa metodologia de ensino é eficaz para reforçar conhecimentos, garantir a segurança do paciente e reduzir os riscos de erros associados a iatrogenias. Ao final da cena, ocorre o *debriefing*, contribuindo para a consolidação do aprendizado^(1)^. Em 2014, surgiram diferentes abordagens, como a prática deliberada em ciclos rápidos (PDCR). Essa metodologia inovadora consiste em dividir o caso clínico simulado em ciclos, que são repetidos pelo aluno até que a competência necessária seja adquirida^(3-5)^. O *feedback* é realizado por ciclos, utilizando um método de ensino com pausas rápidas para corrigir erros e reforçar o aprendizado com base no desempenho do estudante^(6,7)^. É importante ressaltar a variação no método de reforçar o conhecimento adquirido. Na simulação clínica, é adotado o *debriefing* para analisar as situações durante a execução do cenário e incentivar melhorias, sem interromper a atividade. Já na PDCR, o *feedback* é dado ao final de cada ciclo do cenário, possibilitando ao aluno praticar e aprimorar suas habilidades até alcançar o nível desejado dentro de cada ciclo^(8-11)^.

A simulação clínica é uma excelente ferramenta de aprendizado, pois proporciona uma experiência prática antecipada da profissão, possibilitando aos estudantes associar a teoria aprendida em sala de aula com a prática clínica. Isso contribui significativamente para a retenção de conhecimento, além de melhorar o desenvolvimento de habilidades, liderança, tomada de decisão e trabalho em equipe^(10-12)^. Estudos também demonstraram que a PDCR contribui para o aumento do conhecimento e da autoconfiança, e aprimoramento das habilidades dos indivíduos^(13-16)^.

Durante o período de formação na área da saúde, muitos estudantes encontram dificuldades em ter experiência prática em diversas situações clínicas, incluindo o atendimento a pacientes com suspeita de acidente vascular cerebral (AVC). No Brasil, no ano de 2020, foram registrados 99.010 óbitos decorrentes dessa doença e, em 2022, voltou a ser a principal causa de morte no país^(17)^.

A simulação clínica e PDCR são técnicas que podem contribuir no aprimoramento das habilidades necessárias para lidar com pacientes que apresentem sinais e sintomas de AVC. É uma enfermidade capaz de levar à morte ou deixar sequelas físicas e mentais, restringindo suas tarefas diárias, prejudicando a qualidade de vida e eficiência, e acarretando despesas extras com saúde devido às complicações^(18)^. Ainda, é uma das principais causas de morte e incapacidade, e diante do seu impacto econômico/social, faz-se necessário capacitar os discentes para identificação precoce dos sinais e sintomas, de modo a possibilitar um desfecho favorável ao paciente. O tratamento é dependente do tempo, ou seja, a identificação precoce é fundamental para um desfecho mais favorável.

Nesse contexto, recomenda-se um cenário de alta fidelidade para capacitação dos discentes no reconhecimento do AVC e implementação das ações iniciais. O desenvolvimento dessa competência é complexo, pois envolve tomada de decisão, resolução de problemas, comunicação e trabalho em equipe^(17,18)^, sendo um desafio para educadores escolher a melhor estratégia pedagógica para alcançar os objetivos propostos para a atividade. Dessa maneira, considera-se este estudo importante por subsidiar essa escolha pelos facilitadores, proporcionando um planejamento mais assertivo e efetivo.

## OBJETIVOS

Comparar a simulação clínica com a PDCR na aquisição de conhecimento, na percepção sobre a experiência e no *design* do cenário no atendimento ao paciente com suspeita de AVC.

## MÉTODOS

### Aspectos éticos

Esta pesquisa foi aprovada pelo Comitê de Ética em Pesquisa da Universidade Federal de São Paulo. Conforme a Resolução brasileira n^o^ 466/2012, todas as normas éticas e legais referentes às pesquisas com seres humanos foram respeitadas, e o Termo de Consentimento Livre e Esclarecido foi obtido de todos os indivíduos envolvidos no estudo por meio escrito. O ensaio clínico foi homologado através do número U1111-1295-1246 no Registro Brasileiro de Ensaios Clínicos.

### Desenho, período e local do estudo

Trata-se de ensaio clínico randômico, prospectivo, controlado, com alocação aleatória, seguindo as descrições de acordo com o *CONsolidated Standards Of Reporting Trials* ^(19)^. A coleta de dados ocorreu entre os anos de 2022 e 2024, e foi realizada no centro de simulação de uma universidade pública, situada na cidade de São Paulo, SP, Brasil.

### População, critérios de inclusão e exclusão

Foram convidados todos os graduandos regularmente matriculados no último ano do curso de graduação em enfermagem nos anos de 2022 e 2023. Foram incluídos os graduandos que participaram da aula expositiva dialogada sobre assistência de enfermagem ao paciente com suspeita de AVC no serviço de emergência, com duração de 40 minutos. Foram excluídos os graduandos ausentes no dia da coleta de dados.

### Protocolo do estudo

A simulação com paciente padronizado (SCPP) e a PDCR foram aplicadas pela pesquisadora aos participantes a partir do cenário validado para atendimento ao paciente em suspeita de AVC no serviço de emergência. O cenário envolvia o atendimento a um paciente adulto na sala de emergência, com quadro sugestivo de AVC. O paciente do sexo masculino, 65 anos de idade, chega ao serviço de emergência com dificuldades para deambular, hemiparesia e comunicação verbal lentificada. Esperava-se que os grupos iniciassem as avaliações dos sintomas e histórico do paciente, e dessem as devidas orientações, aplicação de escalas, monitorização e coleta de exames de sangue, finalizando com o encaminhamento para o exame de imagem. O grupo SCPP tinha dez minutos para desenvolver o cenário e, ao término, foram identificados pontos positivos e os de melhoria, de acordo com os *guidelines* (*debriefing* - sugestivo de 20 a 30 minutos), e o grupo PDCR levou em média de 60 a 70 minutos para a conclusão dos ciclos. O cenário da PDCR foi dividido em quatro ciclos, que contemplaram os objetivos esperados para cada fase. As ações de correção e sugestões de melhores condutas foram feitas durante os ciclos, e influenciaram a decisão de permitir o aluno percorrer todos os ciclos (*feedback*)^(20)^.

Para garantir a alocação dos discentes, foram confeccionados envelopes numerados, opacos e lacrados. Metade deles continha um papel com a descrição do grupo SCPP, e a outra, a descrição do grupo PDCR, que foram colocados dentro de um papel cartão dobrados para garantir a invisibilidade do texto a todos os participantes envolvidos e da pesquisadora, que conduziu a simulação em todos os grupos. Todos os envelopes foram colocados em uma caixa fosca, com a abertura na tampa superior, pela qual os participantes retiravam antes de iniciar a atividade simulada^(21)^. No grupo SCPP, ainda foram subdivididos em dois grupos: simulação atuando (o aluno realiza o atendimento no cenário); e simulação observando (o aluno observa o grupo que realiza o atendimento no cenário).

O instrumento para avaliação do conhecimento foi validado por um grupo de especialistas e aplicado antes e depois das duas estratégias educacionais. Esse foi composto de nove perguntas objetivas, com quatro alternativas para resposta, tendo apenas uma alternativa correta. As perguntas versavam sobre sinais e sintomas, fatores de risco, escalas de avaliação, e cuidados específicos do paciente com suspeita de AVC.

Imediatamente após as práticas simuladas (SCPP e PDCR), a fim de avaliar a opinião dos estudantes sobre o cenário e a experiência, aplicaram-se a Escala de *Design* da Simulação e o Questionário de Práticas Educacionais (ambos validados para a língua portuguesa)^(22,23)^. O padrão de resposta para ambos questionários foi do tipo Likert de cinco pontos, correspondendo: 1 - Discordo totalmente da afirmação; 2 - Discordo da afirmação; 3 - Indiferente; 4 - Concordo com a afirmação; 5 - Concordo totalmente com a afirmação.

### Análise dos resultados e estatística

Foi realizada análise estatística descritiva (média, mínimo e máximo e desvio padrão) do resultado da aplicação da Escala de *Design* da Simulação e do Questionário de Práticas Educacionais, assim como de cada item e de seus fatores correspondentes. Para fins de tratamento estatístico, o banco de dados foi estruturado no Python 3.9 utilizando a biblioteca Pandas. Foi realizado o teste de Shapiro-Wilk, identificando-se ausência de normalidade, por isso foram utilizados os testes não paramétricos. O teste de Wilcoxon foi utilizado para comparabilidade entre os grupos pareados antes e depois do método de abordagem (média de acertos no prée pós-teste e comparação das respostas dos grupos ao Questionário de Práticas Educacionais) após intervenção educativa, utilizando a alternativa da hipótese “*less*”. A fim de comparar as diferentes estratégias educacionais, foi utilizado o teste U de Mann-Whitney. O nível de significância utilizado como parâmetro foi de 5%.

## RESULTADOS

Foram incluídos 134 estudantes regularmente matriculados da quarta série da graduação em enfermagem. Os estudantes foram divididos através de alocação aleatória em dois grupos: 66 estudantes do grupo SCPP; e 68 estudantes do grupo PDCR. No grupo SCPP, os alunos foram redistribuídos em dois subgrupos: 33 alunos no grupo atuando; e 33 no grupo observando. Em relação ao conhecimento, houve melhora das médias das notas pós-atividade em relação às notas pré-teste, independentemente da estratégia utilizada: PDCR ou SCPP (P<0,001) (Tabela 1).

**Tabela 1:** Comparação da média de acertos no prée pós-teste entre os grupos simulação paciente padronizado e prática deliberada em ciclos rápidos no prée pós-teste, São Paulo, São Paulo, Brasil, 2023 (N=134)

	Pré-teste		Pós-teste		Valor de *p* ^*^
	Média (Desvio Padrão)	Mínimo-Máximo	Média (Desvio Padrão)	Mínimo-Máximo	
Grupo simulação paciente padronizado (n = 66) Grupo prática deliberada em ciclos rápidos(n=68)	7,76 (1,28) 7,96 (1,21)	3,00 - 9,00 3,00 - 9,00	8,44 (0,84) 8,66 (0,63)	5,00 - 9,00 7,00 - 9,00	p<0,001 p<0,001

* *Valor de p - Probabilidade de significância utilizando o teste de Wilcoxon.*

Quando comparado o aumento de conhecimento no précom o pós-teste, independentemente da estratégia utilizada, o grupo PDCR apresentou um ganho de 51,47% na somatória em número de acertos através do questionário aplicado para avaliação do conhecimento logo depois da prática simulada (pós-teste), enquanto o grupo SCPP teve aumento de 46,97%, ou seja, houve uma melhora estatisticamente significante (p< 0,001) como resultado no pós-teste das duas estratégias de simulação. Porém, quando comparadas as duas estratégias, não houve diferença entre os grupos (p= 1,0) (Tabela 1).

Houve melhora do conhecimento dos participantes entre os momentos avaliados (pré-teste e pós-teste), independentemente da alocação no grupo, atuando no atendimento simulado com o uso de paciente padronizado (n=33) ou no grupo observando (n=33) (p<0,001). A melhor média de conhecimento foi a do grupo simulação atuando (8,64) (Tabela 2). Porém, quando comparados os dois grupos (atuando e observando), não houve diferença estatística entre eles (p=0,76).

**Tabela 2:** Comparação da média de acertos no prée pós-teste entre os grupos (simulação atuando e simulação observando), São Paulo, São Paulo, Brasil, 2023 (N=66)

	Pré-teste		Pós-teste		Valor de *p* ^*^
	Média (Desvio Padrão)	Mínimo-Máximo	Média (Desvio Padrão)	Mínimo-Máximo	
Simulação atuando (n= 33) Simulação observando (n=33)	7,97 (1,17) 7,55 (1,35)	4,00 - 9,00 3,00 - 9,00	8,64 (0,59) 8,24 (0,99)	7,00 - 9,00 5,00 - 9,00	p<0,001 p<0,001

* *Valor de p - Probabilidade de significância utilizando o teste de wilcoxon.*

Com relação à análise da Escala de *Design* da Simulação e do Questionário de Práticas Educacionais, as duas estratégias educacionais apresentaram para todos os fatores, bem como para as escalas como um todo, moda de 5 e médias superiores a 4,2, demonstrando uma avaliação muito positiva pelos estudantes para ambas as estratégias (Tabelas 3 e 4).

**Tabela 3:** Comparação das respostas dos grupos simulação com paciente padronizado e prática deliberada em ciclos rápidos ao Questionário de Práticas Educacionais, São Paulo, São Paulo, Brasil, 2023 (N=134)

Fatores	Grupo SCPP^*^					Grupo PDCR^†^					Valor de *p* ^¶^
	Média	DP^‡^	Mín^§^	Máx^||^	Moda	Média	DP^‡^	Mín^§^	Máx^||^	Moda	
Aprendizagem ativa	4,74	0,51	2	5	5	4,80	0,44	1	5	5	p=0,02
Colaboração	4,64	0,73	2	5	5	4,36	0,92	1	5	5	p=0,004
Maneiras diferentes de aprendizagem	4,68	0,49	3	5	5	4,80	0,39	4	5	5	p=0,03
Altas expectativas	4,72	0,56	2	5	5	4,86	0,34	4	5	5	p=0,05
Média Global	4,72	0,54	2	5	5,00	4,75	0,53	1	5	5,00	p=0,05

* *SCPP - Simulação com paciente padronizado; †PDCR - Prática deliberada em ciclos rápidos; ‡DP - Desvio padrão; §Mín - Mínimo; ||Máx - Máximo; ¶Valor de p - Probabilidade de significância utilizando teste U de Mann-Whitney.*

**Tabela 4:** Comparação das respostas dos grupos simulação com paciente padronizado e prática deliberada em ciclos rápidos em relação à Escala de *Design* da Simulação, São Paulo, São Paulo, Brasil, 2023 (N=134)

Fatores	Grupo SCPP^*^					Grupo PDCR^†^					Valor de *p* ^¶^
	Média	DP^‡^	Mín^§^	Máx^||^	Moda	Média	DP^‡^	Mín^§^	Máx^||^	Moda	
Objetivos e informações	4,79	0,44	2	5	5	4,83	0,40	2	5	5	p=0,13
Apoio	4,68	0,63	2	5	5	4,84	0,41	2	5	5	p<0,001
Resolução de problemas	4,73	0,52	2	5	5	4,82	0,43	1	5	5	p=0,02
Feedback / Reflexão	4,87	0,37	2	5	5	4,89	0,30	4	5	5	p=0,67
Realismo	4,54	0,61	2	5	5	4,45	0,69	2	5	5	p=0,33
Média Global	4,75	0,51	2	5	5,00	4,80	0,45	1	5	5,00	p=0,03

* *SCPP - Simulação com paciente padronizado; †PDCR - Prática deliberada em ciclos rápidos; ‡DP - Desvio padrão; §Mín - Mínimo; ||Máx - Máximo; ¶Valor de p - Probabilidade de significância utilizando teste U de Mann-Whitney.*

No Questionário de Práticas Educacionais, o grupo PDCR foi mais bem avaliado no fator aprendizagem ativa (p=0,02), ou seja, os itens oportunidade de refletir sobre o recebimento de pistas durante a prática simulada, discutir os objetivos e as ideias com o facilitador, refletir sobre os comentários do facilitador e aprender com os comentários realizados por ele, e o tempo de aprendizagem foram mais produtivos. No fator colaboração, a estratégia de SCPP foi mais bem avaliada, ou seja, os participantes tiveram mais oportunidade de trabalhar com os colegas durante a prática simulada e realizar o trabalho juntos (p=0,004). Em relação ao fator maneiras diferentes de aprendizagem, o grupo PDCR avaliou mais positivamente, pois opinou que a prática trouxe uma abordagem diferente para contribuir em seu aprendizado. Em seu último fator, que aborda altas expectativas, a PDCR foi mais bem avaliada, pois os participantes refletiram que os objetivos foram claros e de fácil compreensão, e que o facilitador comunicou os objetivos a serem alcançados (p=0,05) (Tabela 3)

Em relação à Escala de *Design* da Simulação, a estratégia PDCR foi mais bem avaliada em relação ao apoio, que se refere quando participante tinha suas necessidades reconhecidas durante a atividade simulada (p<0,001), e à resolução de problemas, que avalia as oportunidades de resolução das situações problemas identificados no cenário (p=0,02). Ainda, no resultado da aplicação da escala como um todo, a estratégia PDCR foi mais bem avaliada pelos estudantes (p=0,03) (Tabela 4).

## DISCUSSÃO

### Simulação com paciente padronizado e prática deliberada em ciclos rápidos

O ensino na graduação em enfermagem tem procurado por estratégias educacionais inovadoras para aquisição das competências profissionais^(24,25)^. Estudos ratificam o achado, demostrando que a simulação é benéfica para a construção do conhecimento de uma forma controlada e segura, assim como para desenvolver a capacidade da resolução de problemas, e oportuniza o recebimento de *feedbacks* sobre a sua atuação, o que contribui para melhorar o seu desempenho^(22,23)^. Há evidências também de que a PDCR aumenta os níveis de conhecimento e autoconfiança, melhorando as habilidades, o que potencializa a prática^(26,27)^.

Estudo^(28)^ realizado com 58 graduandos do curso de enfermagem, que avaliou a eficácia da simulação clínica no ensino de banho no leito, demonstrou que os estudantes que participaram da simulação obtiveram melhor desempenho no pós-teste imediato e tardio. Outro estudo experimental com a temática de administração de medicamentos, realizado com 85 acadêmicos de enfermagem, do tipo prée pós-teste, obteve resultado semelhante^(29)^.

Estudos têm demonstrado a efetividade da PDCR para capacitação em reanimação cardiopulmonar, tendo como resultados as compressões de maior qualidade, tempo mais rápido para iniciar compressões e desfibrilação, inclusive comparando com os treinamentos realizados pela *American Heart Association* ^(30-32)^.

Os resultados foram semelhantes a outro estudo realizado em Ruanda, que comparou as duas estratégias, em que 51 médicos residentes pediátricos foram aleatoriamente designados para o grupo PDCR ou simulação tradicional. Houve aumento de 21% no desempenho prée pós-teste em ambos os grupos, mas não houve diferença entre os grupos^(33)^.

Entretanto, estudo randômico realizado com 76 médicos do Programa de Pós-Graduação em Medicina de Emergência do Hospital Israelita Albert Einstein encontrou resultado discordante quando comparou o desempenho dos participantes na simulação clínica e na PDCR durante reanimação cardiopulmonar. A estratégia PDCR foi associada a uma fração de compressão torácica significativamente maior e tempo significativamente menor entre o reconhecimento do ritmo e a desfibrilação^(34)^.

### Simulação atuando e simulação observando

Os resultados de revisão sistemática que incluiu nove artigos constataram que o papel do observador na simulação teve resultados de aprendizagem iguais ou até melhores do que aqueles que atuaram nos cenários^(35,36)^. Esse fenômeno pode ser explicado pelos princípios da aprendizagem vicária, descrita por Bandura^(37)^, segundo os quais os indivíduos podem adquirir novos conhecimentos, habilidades e atitudes por meio da observação do comportamento de outros, sem a necessidade de vivenciar diretamente a experiência. Outra pesquisa que avaliou a satisfação dos estudantes concluiu que não houve diferença significante entre os papéis desempenhados na simulação (atuante ou observador)^(38)^.

A realização de cenários de simulação com um rigor metodológico, valorizando cada etapa do processo, incluindo todos os participantes, independentemente de seu papel, desde o *briefing* até o *debriefing*, é essencial para garantir que a aprendizagem ocorra da forma mais efetiva possível. Esse achado ratifica o resultado de revisão sistemática que incluiu nove artigos, na qual foi constatado que o papel do observador na simulação teve resultados de aprendizagem iguais ou até melhores do que aqueles que atuaram nos cenários^(35,36)^. Outra pesquisa que avaliou a satisfação dos estudantes concluiu que não houve diferença significante entre os papéis desempenhados na simulação (atuante ou observador)^(38)^.

A realização de cenários de simulação com um rigor metodológico, valorizando cada etapa do processo, incluindo todos os participantes, independentemente de seu papel, desde o *briefing* até o *debriefing*, é essencial para garantir que a aprendizagem ocorra da forma mais efetiva possível.

### Questionário de Práticas Educacionais e Escala de *Design* da Simulação

No Questionário de Práticas Educacionais, a PDCR foi mais bem avaliada em três fatores, mas somente foi pior no fator colaboração, o que pode ser justificado pelo fato de que, na estratégia PDCR, os estudantes executaram o atendimento ao paciente com suspeita de AVC de maneira individual e, no grupo SCPP, realizavam os atendimentos em duplas.

As atividades educacionais estão presentes nas abordagens e técnicas de ensino^(39)^. As práticas educacionais eficazes envolvem seguir diretrizes que contribuam para aprendizagem, como incentivar a interação entre estudante e professor, promover a cooperação entre os estudantes e aprendizagem ativa, fornecer *feedback* imediato, dedicar tempo para estudo, estabelecer expectativas elevadas, e respeitar as habilidades dos estudantes e suas maneiras de aprender^(40)^.

Para que a aprendizagem seja efetiva, é essencial que o aluno tenha a chance de debater e expandir seus conhecimentos com base em seus aprendizados prévios - conceitos, experiências, entre outros - e compartilhá-los com o facilitador, outros estudantes e colaboradores^(41)^. A participação ativa do aluno em cenários simulados permite a ele analisar criticamente e avaliar diversas situações práticas. Essa reflexão é fundamental para aprimorar sua prática profissional^(41,42)^. Além disso, quanto mais ativamente o aluno participa do processo de aprendizagem, melhor ele é capaz de assimilar as informações fornecidas^(42)^; é o que evidencia a avaliação dos estudantes nos fatores aprendizagem ativa e altas expectativas sobre a estratégia PDCR. É importante a participação do aluno no processo de compartilhamento dos objetivos elencados, destacando a importância na interface de ensino e aprendizado. Quanto aos comportamentos dos participantes (facilitador e estudantes), espera-se que apresentem atributos de confidencialidade, compaixão, compromisso, colaboração, honestidade, respeito mútuo e envolvimento no processo de ensino e aprendizagem^(41)^.

A análise do *design* nas práticas simuladas evidencia a necessidade de aprimoramentos que potencializem a efetividade do cenário como estratégia de ensino-aprendizagem. Nesse processo, o papel do facilitador mostra-se não só relevante, mas essencial para a construção do conhecimento. A literatura^(39-42)^ aponta que a atuação do facilitador transcende a função operacional de condução da simulação, assumindo um papel estratégico no desenvolvimento do pensamento crítico, no raciocínio clínico, no julgamento profissional e na transposição do conhecimento teórico para a prática assistencial. Em uma perspectiva construtivista, o facilitador se configura como um agente catalisador da aprendizagem significativa, ao promover reflexões, questionamentos e *feedbacks* direcionados, que estimulam o protagonismo do estudante no processo de aprendizagem.

### Limitações do estudo

As limitações deste estudo incluem a realização dos testes em um único centro de simulação e a participação exclusiva de estudantes de enfermagem. Além disso, embora houvesse um *checklist* objetivo para guiar a avaliação das ações esperadas nos cenários (SCPP e PDCR), a aplicação dessas estratégias pela própria pesquisadora pode ter introduzido um viés.

### Contribuições para as áreas da enfermagem, saúde ou políticas públicas

A avaliação positiva das duas estratégias demonstra que o uso de metodologias ativas na área da saúde, mesmo em instituições com recursos limitados, pode viabilizar o aprendizado e aumentar as chances de reconhecimento e tratamento precoce de pacientes com suspeita de AVC. Isso contribui para melhores desfechos clínicos e redução de custos ao sistema de saúde. O estudo reforça que tanto a SCPP quanto a PDCR são estratégias eficazes no desenvolvimento de competências em enfermagem. A PDCR apresenta ainda vantagens específicas no engajamento dos estudantes e no *design* pedagógico, aspectos importantes para decisões curriculares.

## CONCLUSÕES

A PDCR e SCPP se mostraram estratégias igualmente eficientes para melhorar o conhecimento sobre atendimento inicial à paciente vítima de AVC. A partir da avaliação da experiência simulada pelos estudantes, mediante a Escala de *Design* da Simulação, a estratégia PDCR obteve melhor avaliação, quando comparada ao grupo SCPP (p=0,03).

Na comparação entre os subgrupos da SCPP (atuando e observando), não houve diferença na aquisição de conhecimentos. Isso indica que, independentemente da posição ocupada (atuando ou observando), ambos os papéis foram eficazes para melhorar o conhecimento sobre o atendimento inicial à vítima de AVC.

## Data Availability

Os dados da pesquisa estão disponíveis no corpo do artigo.
